# Association of *SORD* mutation with autosomal recessive asymmetric distal hereditary motor neuropathy

**DOI:** 10.1186/s12920-022-01238-4

**Published:** 2022-04-18

**Authors:** Majed Alluqmani, Sulman Basit

**Affiliations:** 1 College of Medicine, Taibah University Medina, Medina, Saudi Arabia; 2 Center for Genetics and Inherited Diseases, Taibah University Medina, Medina, Saudi Arabia

**Keywords:** Hereditary neuropathy, Nerve conduction, Electromyography, *SORD* mutation

## Abstract

**Background:**

The aim of this study was to identify the underlying genetic defect in a family segregating autosomal recessive asymmetric hereditary motor neuropathy (HMN). Asymmetric HMN has not been associated earlier with *SORD* mutations.

**Methods:**

For this study, we have recruited a family and collected blood samples from affected and normal individuals of a family. Detailed clinical examination and electrophysiological studies were carried out. Whole exome sequencing was performed to detect the underlying genetic defect in this family. The potential variant was validated using the Sanger sequencing approach.

**Results:**

Clinical and electrophysiological examination revealed asymmetric motor neuropathy with normal nerve conduction velocities and action potentials. Genetic analysis identified a homozygous mononucleotide deletion mutation (c.757delG) in a *SORD* gene in a patient. This mutation is predicted to cause premature truncation of a protein (p.A253Qfs*27).

**Conclusions:**

Interestingly, the patient with homozygous *SORD* mutation demonstrates normal motor and nerve conduction velocities and action potentials. The affected individual describes in this study has a unique presentation of asymmetric motor neuropathy predominantly affecting the right side more than the left as supported by the clinical examination. This is the first report of *SORD* mutation from Saudi Arabia and this study further expands the phenotypic spectrum of *SORD* mutation.

## Introduction

Distal hereditary motor neuropathy (dHMN) is a clinically and genetically heterogeneous disorder affecting the muscles of distal limbs. Individuals with dHMN experience progressive weakness and atrophy of the muscles of the distal limbs [1]. In dHMN, generally, there is no involvement of sensory neurons, however, in some cases minimal involvement of sensory neurons is reported [2]. Based on the inheritance pattern and the clinical features, dHMN has been divided into seven subgroups [3]. Autosomal recessive dHMN may appear early in life with mild as well as severe clinical features. dHMN and Charcot-Mare-Tooth (CMT) diseases are clinically and genetically overlapping disorders and in some cases, they share the underlying genetic defects [4, 5]. For instance, mutations in *HSPB1*, *IGHMBP2*, and *DYNC1H1* cause both CMT and dHMN [1, 6–15]. Moreover, mutations in the sorbitol dehydrogenase (*SORD*) gene have recently been associated with the autosomal recessive form of Charcot-Mare-Tooth disease type 2 (CMT2) and dHMN [1, 8, 16, 17]^.^ Although, a clinical and genetic overlap exists between CMT2 and dHMN, however, motor nerves are predominantly or exclusively involved in dHMN [2].

We recruited a family segregating autosomal recessive dHMN. Clinical and genetic analysis was performed and a homozygous nonsense mutation in the *SORD* gene (c.757delG; p.Ala253GlnfsTer27) was identified. The mutation has been shown to cause a complete loss of SORD protein resultantly an increased sorbitol level in the cells.

## Methods

### Ethical approval

All study protocols were approved by the scientific research ethics committee of the College of Medicine, Taibah University Medina. The ethical approval ID is 036-1441. All experimental work was performed in accordance with the declaration of Helsinki. Written informed consents were obtained from all the participants for genetic analysis of the DNA samples and publication of the genetic data.

### Genetic studies

**Fig. 1:**
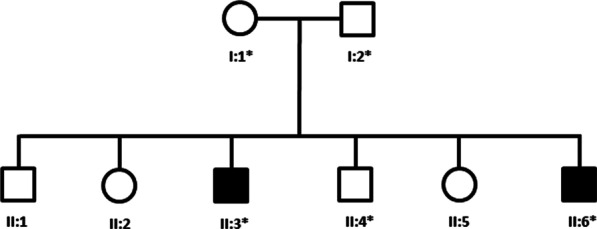
Pedigree of a family recruited for this study. A star indicates the family member whose DNA was available for the sequencing. Females are represented by circles and males by squares, those individuals with solid symbols have hereditary motor neuropathy while those with clear symbols are unaffected

Genomic DNA was extracted from the peripheral blood of a proband (II:3), unaffected parents (I:1 and I:2), a healthy individual (II:4), and an affected sibling (II:6) (Fig. 1). The complete coding regions (~ 22,000 genes) of the human genome was captured by xGen Exome Research Panel v2 (Integrated DNA Technologies, Coralville, Iowa, USA). The captured region of the human genome was sequenced with NovaSeq 6000 system (Illumina, San Diego, CA, USA). The raw sequencing data analysis, including alignment to the GRCh37/hg19 human reference genome, variant calling, and annotation, was conducted with open-source bioinformatics tools and in-house software. A variant interpretation was performed with in-house software to prioritize variants based on ACMG guidelines considering the phenotype of the patient. This system has three major steps; variant filtration, classification, and similarity scoring for patient’s phenotype. The following steps were used to filter and prioritize candidate variants. First, gnomAD (http://gnomad.broadinstitute.org/) as a population genome database were used for estimating allele frequency. Common variants with a minor allele frequency of > 5% were filtered out in accordance with BA1 of the ACMG guideline. Second, scientific literature and disease databases including ClinVar (https://www.ncbi.nlm.nih.gov/clinvar/) and UniProt (https://www.uniprot.org/) were searched and evidence data on the pathogenicity of variants was extracted. The pathogenicity of each variant was evaluated according to the recommendations of the ACMG guideline. Third, the clinical features of the patient were coded as standardized human phenotype ontology terms (https://hpo.jax.org/) and accessed to measure the similarity with each of ~ 7000 rare genetic diseases (https://omim.org/ and https://www.orpha.net/consor/cgi-bin/index.php). The similarity score was calculated for the patient’s phenotype and the prioritized variants. Finally, medical geneticists manually evaluated the candidate variants and associated diseases. The variant of interest was Sanger sequenced in the proband, both parents, a healthy family member, and an affected sibling (Fig. 1).

## Results

### Clinical description of the patient

A 26 years old patient (II:3) presented with a history of progressive right leg weakness was examined. Initially, he was diagnosed with a distal myopathy. He has been complaining of right knee pain and limping to the right side after long-distance walks. He was free of any other neurological symptoms including muscle cramping, abnormal twitching, muscle fasciculation, and tingling or numbness. He was examined by a consultant neurologist. Motor nerve conduction (MNC) and sensory nerve conduction (SNC) studies have been performed. Moreover, electromyography (EMG) was also carried out. The proband has informed us about another sibling (II:6) with a similar clinical picture.

On physical examination, the patient (II:3) was found to have a clear picture of distal neuropathy as evident from a foot deformity, including the common pes cavus, hammer toes, and twisting of the ankle on both sides, more severe on the right side. There was a mild weakness (graded as + 4) on the right foot dorsiflexion and the right ankle reflex was absent. Also, the pinprick and temperature sensation were mildly reduced in the right foot. The posterior column tract examination was intact as well as the gait was within a normal limit (Table 1).

**Table 1:** Clinical features observed in the proband

Clinical features	Comments
Age of onset (years)	23
Age at examination (years)	26
Family history	Brother has the same symptoms
CMT subtype	dHMN
Foot deformities	Pes caves, hammer toes, callosities
Upper-limb weakness	*Normal upper limb examination
Proximal muscle groups	*Lower limb examination: by inspection looks inverted champagne bottle
Distal muscle groups	Bilateral distal weakness including knee flexion and extension and ankle dorsal flexion and planter flexion, however, it is more in right than left
Reduce vibratory sensation	No
Reduced pinprick superficial sensation	Mild in the right foot
Disease severity	Mild
Use of ankle–foot orthoses	No
Other walking aids	No
**Nerve conduction study**	
Reduced motor conduction velocity	No
Reduced sensory action potentials	No
Compound muscle action potential	Reduced
Charcot-Marie-Tooth disease examination score	2
Tendon reflexes	Absent in ankle

#### Motor nerve conduction (MNC) studies

Bilateral tibial MNC studies of the abductor halluces revealed a normal distal nerve latency, compound muscle action potential (CMAP) amplitude, and conduction velocity. Moreover, minimal F wave latency was also in the normal range. Right and left peroneal MNC studies recording from the extensor digitorum brevis revealed normal distal motor latency, CMAP amplitude (at 2.3 mV), and conduction velocity. However, comparing the peroneal nerve amplitude of the right side with the left side, the right side amplitude was 50% less than the left side, although, both were within the normal limits.

#### Sensory nerve conduction (SNC) studies

Bilateral superficial peroneal sensory nerve conduction studies reveal normal peak latencies, sensory nerve action potentials (SNAP) amplitudes, and conduction velocities. Moreover, bilateral sural SNC studies reveal normal peak latencies, SNAP amplitudes and conduction velocities.

#### Needle electromyography (EMG)

Needle examination of the right first dorsal interosseous (FDIO) space, deltoid and extensor digitorum communis (EDC) revealed normal insertional activity with no spontaneous activity. Motor unit potentials were broad and of high amplitude with a slightly reduced interference pattern. Moreover, the right medial gastrocnemius and left tibialis anterior revealed normal insertional activity. 2 + fibs (fibrillation/positive sharp waves) and positive sharp waves with occasional runs of complex repetitive discharges (CRDs) were observed. The right tibialis anterior, and right vastus lateralis revealed normal insertional activity without any spontaneous activity. The motor unit potentials were of high amplitude (polyphasic) and of broad duration with a slightly reduced interference pattern (Table 2).

**Table 2:** Motor (MNC) and sensory nerve conduction (SNC) studies. (a) Motor nerve conduction studies. (b) Sensory nerve conduction studies

Nerve	Lat (ms)	Amp (mV)	CV (m/s)	F Lat (ms)
(a)				
**Peroneus motor right**				
Ankle-EDB	4.33	2.7		49.0
Fib. Hand-Ankle	10.6	2.3	47.8	
Pop-Fib. head	12.6	2.3	50.0	
**Peroneus motor left**				
Ankle-EDB	4.27	4.0		46.6
Fib. Hand-Ankle	10.9	3.4	46.0	
Pop-Fib. head	12.4	3.1	63.3	
**Tibialis motor right**				
Med. Mal –Abd hal	3.06	5.1		48.9
Bl. Knee-Med. mal	10.6	3.4	49.1	
**Tibialis motor left**				
Med. Mal Abd hal	3.11	6.3		46.6
Bl. Knee-Med. mal	10.6	5.6	49.4	

Nerve	Peak Lat (ms)	Amp (uV)	CV (m/s)	XXXX
**(b)**				
**Suralis sensory right**				
Mid. Lower leg—Ext Saph	2.56	16.4	57.9	
**Suralis sensory left**				
Mid. Lower leg—Ext Saph	2.79	11.2	53.4	

Overall it is concluded from the electrophysiological studies that the patient has active denervation in the right lower extremities. His clinical presentation is asymmetrical. In general, the patient has a CMT neurological score of 3 based on the physical and neurophysiological examination [18].

### A frameshift variant in the *SORD* gene was identified

A high-quality exome data with more than 100 × coverage was obtained (Table 3). Exome data analysis including variants annotation, filtration, and prioritization identified a homozygous deletion variant (c.757del) in the *SORD* gene. CG dinucleotides were found deleted which consequently lead to a frameshift in the protein coding sequence. This frameshift is predicted to cause premature protein truncation. This variant is classified as pathogenic according to the recommendation of the ACMG/AMP guideline.

**Table 3:** Target region coverage statistics

Mean depth (x)	Target base pairs covered (%)				
	≥ 1x	≥ 5x	≥ 10x	≥ 20x	≥ 50x
114.48	99.4	99.2	99.1	99.0	95.2

### Sanger validation of variant

DNA of the proband, an affected sibling, both parents as well as an unaffected member of the family was PCR amplified using primer pair flanking *SORD* variant. The amplicons were sequenced using the Sanger approach. BioEdit sequence alignment tool was used to align the patient sequence reads with the reference sequence. The patient and an affected sibling were found homozygous for the deletion variant. However, both parents were found heterozygous and an unaffected member of the family has a wild-type sequence.

## Discussion

dHMN accounted for a small proportion of inherited peripheral neuropathy. Considering the wide phenotypic and genetic heritability the diagnostic rate in dHMN ranges from 14 to 39% [1, 5, 11, 19, 20]. Low diagnostic rate in dHMN indicates the presence of an unidentified mutation in novel candidate genes. Large scale studies are needed to identify new causative mutations which would ultimately help in delineating the molecular mechanism underlying dHMN pathogenesis.

We studied a family segregating dHMN in an autosomal recessive manner. Electrophysiological studies including MNC, SNC, and EMG revealed velocities and amplitudes in the normal range. Clinically patient is showing abnormal features specifically the right side of the body is asymmetrically affected. The proband showed overlapping clinical features of both CMT type 2 and dHMN. However, his neurological phenotype was asymmetrical. Due to the heterogeneous nature of the disease, we performed whole-exome sequencing and identified a homozygous dinucleotide deletion (c.757delG) in the *SORD* gene. This mutation has recently been reported as the most frequent cause of autosomal recessive hereditary neuropathy [1, 17]. This study supports the hypothesis that the specific *SORD* mutation (c.757delG) is the most common cause of childhood-onset mild form of the autosomal recessive dHMN. This is the first report of *SORD* mutation from Saudi Arabia and broadens the mutation continuum of *SORD* and phenotypic heterogeneity of the dHMN. The specific allele (c.757delG) of *SORD* is wide spread and has been reported by a group from different populations including Chinese, UK, USA, and Turkey [17, 19–21]. This support the notion that this allele is of an ancient origin.

Sorbitol dehydrogenase deficiency with peripheral neuropathy is associated with mutations in the *SORD* gene. To our knowledge, around 16 bi-allelic mutations in the *SORD* gene have been identified [16–20]. SORD-related neuropathy has been reported as one of the most frequent causes of autosomal recessive CMT2 and dHMN [17]. The deletion mutation c.757delG (p.A253Qfs*27), identified in this study, is the only reported variant in SORD-related dHMN [16, 17, 21]. An exception is a Chinese patient with dHMN harboring the compound heterozygous c.404 A > G and c.9081 + G > C mutation [22]. Almost all mutations in *SORD* are predicted to cause loss of function of sorbitol dehydrogenase, which is a key enzyme in sorbitol to fructose conversion. The molecular pathway underlying motor-predominant peripheral neuropathy due to sorbitol dehydrogenase deficiency is not well understood.

## Data Availability

The vcf file of a patient containing whole exome sequencing data has been submitted to the European Variation Archive (EVA). The accession number is PRJEB48950 and the link to data is https://www.ebi.ac.uk/eva/?Study-Browser&browserType=sgv.
